# Microbial characterization of bee pollen from the Vesuvius area collected by using three different traps

**DOI:** 10.1371/journal.pone.0183208

**Published:** 2017-09-21

**Authors:** Gianluigi Mauriello, Annachiara De Prisco, Gennaro Di Prisco, Antonietta La Storia, Emilio Caprio

**Affiliations:** Department of Agricultural Sciences, University of Naples Federico II, Portici, Italy; Seconda Universita degli Studi di Napoli, ITALY

## Abstract

Flower pollen is collected by honeybee foragers, adhered on their rear legs and transported into the hives in the form of pellets. Once in the hives, bee pollen is moisturised with nectar and bee mouth secretions and due to enzymatically modifications it becomes the so-called bee-bread, the protein reservoir of young bees. Bee pollen can be artificially removed from bee legs and collected by using specific systems, the bee pollen traps. Bee pollen is commercialized for human consumption as fresh product and after freezing or drying. Although bee pollen is nowadays largely consumed in developed countries, as food or food supplement according to local legislation, little is known on its safety related to microbiological hazards. In this work, we aimed to characterize for the first time the microbiological profile of Italian bee pollen in fresh, frozen and dried form collected along an entire harvesting season. Moreover, monthly microbiological analyses were performed on frozen (storage at -18°C) and dried (storage at room temperature) bee pollen over a 4 months period. Further aim of this work was the evaluation of the possible impact on production level of three different traps used for pollen collection. Our results on microbial contamination of fresh and frozen bee pollen show that a more comprehensive microbiological risk assessment of bee pollen is required. On the other side, dried pollen showed very low microbial contamination and no pathogen survived after the drying process and during storage.

## Introduction

Nutrition plays an essential role in the health and growth of honeybee (*Apis mellifera* L.) colonies. Flower nectar and pollen provide nutrients for bees, being the former source of energy and the latter of aminoacids and vitamins. Pollen is collected from flowers and moistened by bees with nectar and mouth secretions, becoming “bee pollen”, to be accumulated on *curbicula* of bee rear legs and transported into the hives [1, 2]. Bee pollen is stored in honeycomb where the lactic acid fermentation occurs [3]. This kind of pollen is called "bee bread".

Nowadays, bee pollen is considered a human functional food [4] and it is a growing business for the beekeeping industry. Different types of pollen traps have been designed from the basic concept of scraping pollen off the bees’ legs as they are forced to enter the hive passing through a grid with holes of specific size. Pollen falls in a tray, located under the trap, covered with mesh to prevent retrieval of pollen by the bees [5]. A lot of works describe the chemical composition of bee pollen of different plants and geographical origin [6–9]. Instead, little attention was dedicated to the microbial contamination of bee pollen and to microbiological risks related to its human consumption [10–15]. Furthermore, the availability of microbiogical data is restricted to few countries (e.g. Brazil, Spain, Slovakia). Interestingly, the European Food Safety Authority published a Scientific Report on the risk assessment of multiple stressors on bees, including among stressors the microbial contamination of bee bread [16].

In our opinion, due to the important impact that bee pollen is gaining in the field of human nutrition, an assessment of microbiological aspects in many word areas as well as establishing of guidelines for microbiological standards is of a paramount importance for the assurance of human safety. Bee pollen is mostly commercialized and consumed after being dried to guarantee long-term stability and safety. However, drying treatments, especially when carried out over 40–50°C could affect pollen organoleptic features and polyphenols and flavonoids content [17, 18]. Freezing of bee pollen might be an alternative way to preserve its organoleptic properties and nutritional content, obviously depending on the quality of the initial fresh pollen.

Aim of this work was to elucidate the possible microbiological hazards of bee pollen, that is the first step of a comprehensive risk assessment for this unconventional food product.

## Materials and methods

### Apiary setup

**Fig 1:**
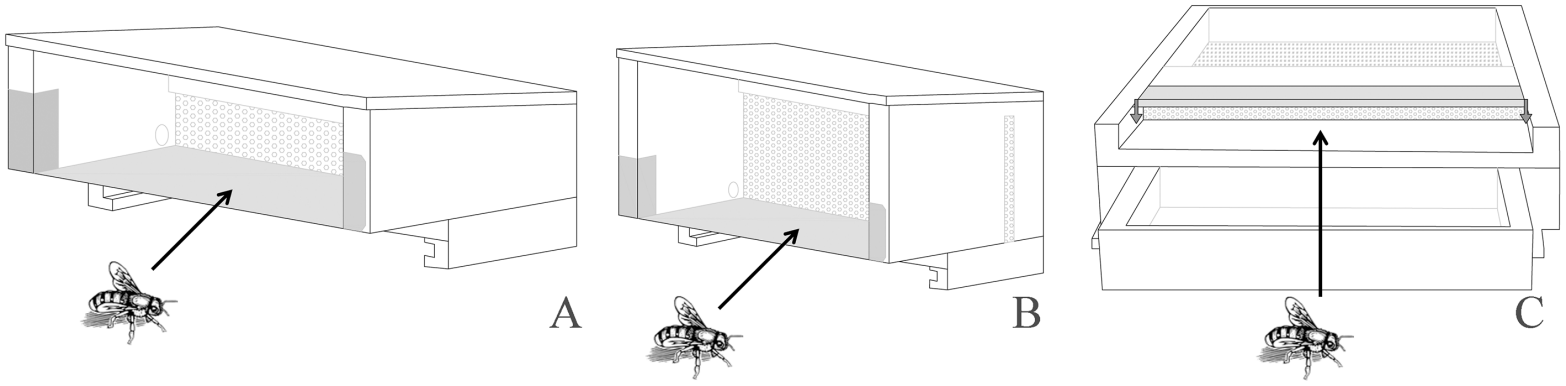
Description and representation of pollen trap systems used in this study. (A) Front trap—FT: this is a small trap that is fastened to the front of a hive with the use of two small hooks and covering the existing entrance. The bees enter into the hive by walking on a metallic mesh that connects the pollen trap to the landing board of the hive. Round shaped holes of the trap strip pollen from bees with minimum damage. Pollen accumulates in a side opening drawer (not showed in the picture). They have the advantage of being easily removed, although their storage capacity is far lower than bottom and wall traps. (B) Wall trap—WT: it is a modified front trap. For its correct positioning it is necessary to apply a change to the natural flight opening of the hive: the latter is occluded with a metal sheet and replaced with three holes created especially in the area immediately above having a diameter of about 5 centimeters. On the new flight opening that is positioned at 2/3 of the beehive the trap will be then applied (for this reason is also called high trap). WT can be mounted on all type of hive and permits that pollen is recovered cleaner and less humid. (C) Bottom trap—BT: this trap is fixed on the bottom of the hive that need to be modified. Bees pass through a series of mesh screens, that remove the pollen from their rear legs. The pollen drops down through the screens into pollen drawer and can be harvested without opening the hive. This pollen trap can virtually occupy all the area of hive to promote good ventilation for dry pollen. The trap can be removed easily when not needed.

**Fig 2:**
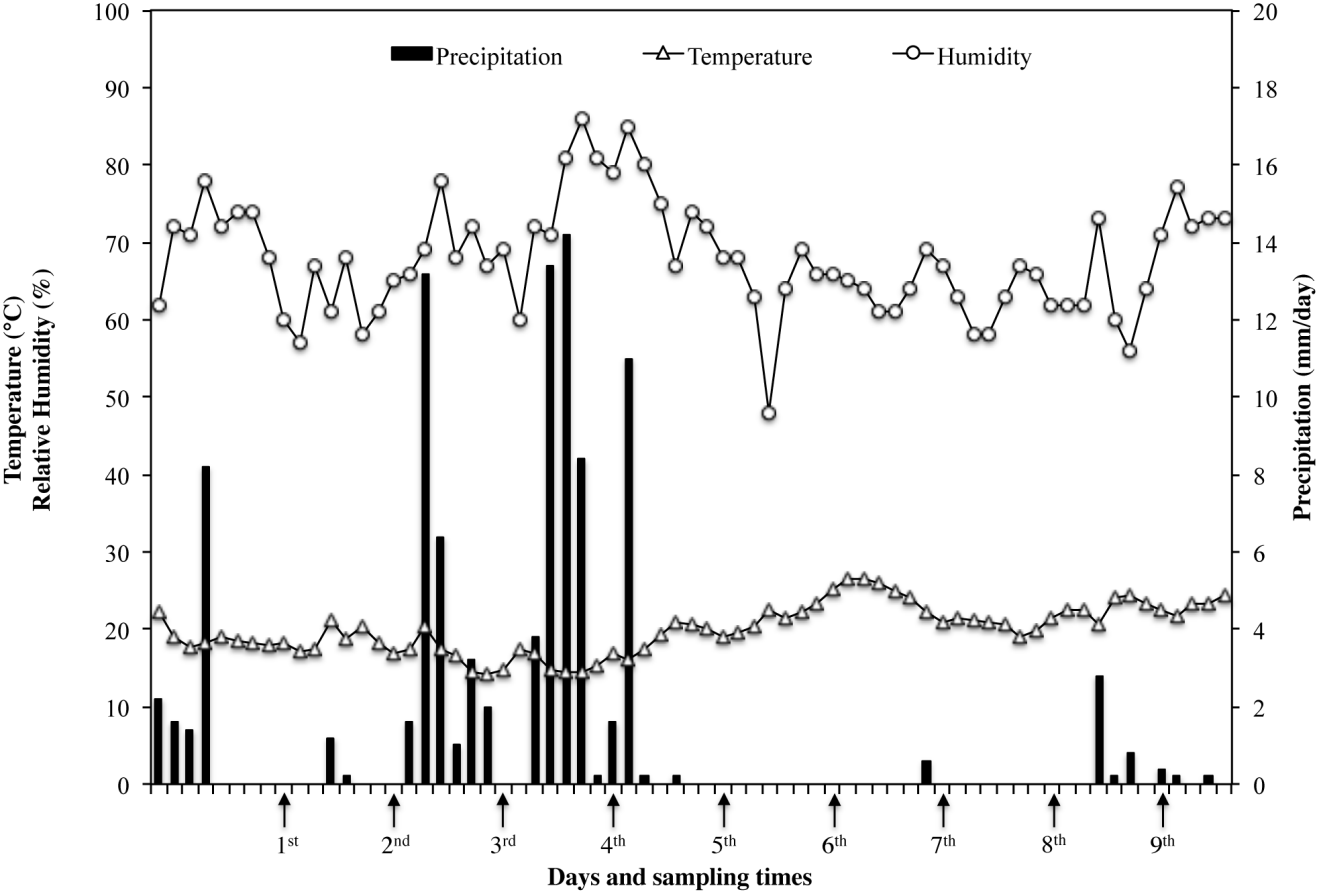
Meteorological data. Data were registered during the pollen collection period by the closest meteorological station of the “Regional Agro-meteorological Center”.

Fifteen honeybee colonies, with queen of the same age, comparable in terms of brood, adult abundance and food storage, were selected for the experiments. The experimental colonies were maintained, following the standard beekeeping techniques, in the Vesuvius area (Campania Region, Italy GPS coordinate lat 40.7946303 long 14.39335010000002) from May to July, covering the entire pollen production time. The study was carried out on a private land and the owner gave his permission to conduct the study on this site. The apiary is registered at the National Registry Office Beekeeping of Ministry of Health with the n. 049NA899. Three different bee pollen traps were used: Front Trap (FT), Wall Trap (WT) and Bottom Trap (BT). Picture and description of each system is reported in Fig 1. Five different colonies were maintained for each type of trap. Furthermore, meteorological data (average temperature, relative humidity and precipitation level) summarized in the graph in Fig 2 were obtained from the official database of “Regional Agro-meteorological Center”, choosing the closest meteorological station.

### Sampling of bee pollen

Bee pollen from each hive was weekly collected during a period of about 2 months (from mid-May to mid-July), for a total of 9 sampling (Fig 2) and rapidly transported in refrigerated conditions. Traps remained mounted on the hives only three days per week to affect less possible honey production. At each sampling time an aliquot of 50 g of bee pollen was collected from each hive. Portable Bunsen burner and sterile equipment were used to avoid microbial contamination during sampling procedures. Aliquots from hives mounting the same type of trap were bulked and, within 24 h at refrigerated temperature, analysed in triplicate as fresh bee pollen. The pollen yield for each type of trap was evaluated by weighting.

At the starting, intermediate and end time of harvesting season, corresponding to the 1^st^, 4^th^ and 9^th^ week of collection, dried (40°C for 16 h) and frozen (-18°C) samples of pollen were prepared from fresh bee pollen. Dried and frozen samples were stored for four months at room temperature (22±2°C) and -18°C, respectively, and monthly analysed for microbial load.

### Culture-dependent microbiological analysis of bee pollen

Fresh, dried and frozen pollen samples were investigated for the following microbiological parameters: mesophilic total viable count (MTVC), yeast and moulds (YM), lactic acid bacteria (LAB), *Enterobacteriaceae*, *Clostridium perfringens*, *Bacillus cereus*, *Escherichia coli*, *Staphylococcus aureus*, *Salmonella* and *Listeria monocytogenes*. According to the ISO 6887–6, 10 g of each bee pollen sample were decimally serially diluted in quarter strength Ringer solution (Oxoid) and used for MTVC, YM, LAB and *Enterobacteriaceae* analysis. MTVC was performed on Standard Plate Count Agar (PCA, Oxoid) at 30°C for 24 h. YM were counted at 22°C for 48 h on Yeast Extract Peptone Dextrose Agar (YPD Agar, Sigma-Aldrich) added of 200 mg/l of chloramphenicol (Sigma-Aldrich) after sterilization. LAB were cultured in de Man Rogosa Sharp (MRS Agar, OXOID) plates incubated at 37°C in anaerobic condition for 48 h. *Enterobacteriaceae* were counted on Violet Red Bile Glucose Agar (VRBGA, Oxoid) at 32°C for 24–48 h. For detection of *B*. *cereus*, *E*. *coli* and *S*. *aureus*, bee pollen samples were five fold serially diluted in quarter strength Ringer solution. *B*. *cereus* was counted at 37°C for 24–48 h on Bacillus cereus Selective Agar Base (Oxoid) supplemented after sterilization with 100,000 IU/l of Polymixin B (Oxoid) and 50 ml/l of Egg Yolk Emulsion (Oxoid). Dilutions of samples were treated before inoculum at 80°C for 10 min in water bath to destroy vegetative cells. *E*. *coli* was counted on Tryptone Bile X-Glucoronide Medium (TBX Agar, Oxoid) at 37°C for 48 h according to the ISO 16649–2. Baird Parker Agar Base (Oxoid) supplemented with 50 ml/l of Egg Yolk Tellurite Emulsion (Oxoid) and incubated at 37°C for 24–48 h was used for detection of *S*. *aureus* colonies. Presence of *Salmonella* was ascertained according to the ISO 6579. Briefly, 25 g of each sample were diluted in 225 ml of Buffered Peptone Water (Oxoid) and incubated for pre-enrichment at 37°C for 20 h. After this period an aliquot of 0.1 ml was transferred in 10 ml of Rappaport-Vassiliadis Broth (Oxoid) and incubated at 37°C for 24–48 h. Finally, 0.1 ml of culture was spread plated on Xylose Lysine Desoxycholate Agar (X.L.D. Agar, Oxoid). *C*. *perfrigens* was detected by diluting 25 g of sample in 225 ml of Buffered Peptone Water (Oxoid) and incubated for pre-enrichment at 37°C for 24 h. Culture was decimally serially diluted in Buffered Peptone Water and 0.1 ml of each dilution was spread plated on Perfrigens Agar Base (Oxoid) added with 12 ml/l of Perfringens Supplement (SFP) and 50 ml/l of Egg Yolk Emulsion (Oxoid) after sterilization at 37°C for 24 h in anaerobic condition. For detection of *L*. *monocytogenes* 25 g of each sample were diluted in 225 ml of Listeria Enrichment Broth added of Listeria Selective Enrichment Supplement (Oxoid) and incubated at 30°C. After 1, 2 and 7 days of incubation undiluted aliquots of 0.1 ml and aliquots diluted 1:10 (v/v) in 0.5% potassium hydroxide were spread plated on Listeria Selective Agar, Oxford formulation (Oxoid) added of the appropriate Listeria Selective Supplement (Oxoid) and aerobically incubated at 37°C for 24–48 h.

Some typical colonies from all growing media, with exclusion of PCA, YPD and VRBGA, were streaked on Tryptone Soy Broth (TSB, Oxoid) supplemented with 5 g/l of Yeast Extract (Oxoid) and 15 g/l of agar (Agar n° 1, Oxoid) and incubated at optimal growth conditions. Resulting cultures were characterised on the base of cell morphology, Gram reaction, catalase and oxidase test. Some cultures responding to typical characteristics of each presumptive species were randomly picked and stored at -18°C in glycerol for further investigations.

### Identification at species level of isolates from bee pollen

Bacterial cultures isolated as above described were identified by 16S rDNA sequencing. DNA extraction was carried out from a single colony by using an InstaGene Matrix (Bio-Rad Laboratories, Hercules, CA) following the conditions described by the supplier. About 25 ng of DNA were used for PCR amplification. Synthetic oligonucleotide primers described by Weisburg *et al*. [19] fD1 (5′-AGAGTTTGATCCTGGCTCAG-3′) and rD1 (5′-AAGGAGGTGATCCAGCC-3′) (*E*. *coli* positions 8–17 and 1540–1524, respectively) were used to amplify the 16S rDNA. PCR mixture was prepared as previously reported [20]. PCR conditions consisted of 30 cycles (1 min at 94°C, 45 sec at 54°C, 2 min at 72°C) plus one additional cycle at 72°C for 7 min as a final chain elongation. The presence of amplicons was verified by agarose (1.5% w/v) gel electrophoresis, at 100 V for 2 h, and bands purified by using a QIAquick gel extraction kit (Qiagen S.p.A., Milan). The DNA sequence was determined by the dideoxy chain termination method by using the primer fD1 [19]. DNA sequence alignment was performed using the National Center for Biotechnology Information (NCBI) online GenBank tools [21]. Sequencing data were deposited in the GeneBank database of the NCBI.

### High Throughput Sequencing

Fresh bee pollen samples collected at 1^st^ sampling time from FT, WT and BT were suspended in quarter strength Ringer solution (Oxoid, Milano, Italy) in 1/5 ratio. Microbial DNA extraction was directly performed from the pellet (12000 g) obtained from 3 ml of the suspension using the Biostic Bacteremia DNA isolation kit (MoBIO Laboratories, Inc. Carlsbad, CA). DNA was quantified by using the Nanodrop Instrument (Spectrophotometer ND-1000, Thermo Fisher Scientific, Milan, Italy) and it was standardized at 60 ng/μl. The microbial diversity was studied amplifying V1–V3 region of the 16S rRNA gene by using primers and PCR condition previously described [22]. PCR samples were purified by the Agencourt AMPure kit (Beckman Coulter, Milan, Italy) and quantified using the Plate Reader (Eppendorf AF2200). The equimolar amplicon pool of PCR templates was used for pyrosequencing by Titanium chemistry on a GS Junior platform (454 Life Sciences, Roche, Monza, Italy) according to the manufacturer instructions.

Raw reads were first filtered according to the 454 processing pipeline. Sequences were then analysed and further filtered by using QIIME 1.9.0 software and a pipeline previously described by De Filippis *et al*. [23]. Briefly, raw reads were demultiplexed and further filtered through the split_library.py script of QIIME. To guarantee a higher level of accuracy in terms of Operational Taxonomic Units (OTUs) detection, the reads were excluded from the analysis if they had an average quality score of lower than 25, if there were ambiguous base calls, if there were primer mismatches and if they were shorter than 300 bp. OTUs defined by a 97% of similarity were picked against the Greengenes database 16S rRNA gene [24]. Any remaining sequences that were of chloroplast or mitochondrial origin were removed.

All of the sequencing data were deposited in the Sequence Read Archive of the NCBI (accession number SRP077038).

### Data analysis

To ascertain statistical differences among the observations a one-way Anova was performed by using Stata v.13 for Mac OSX v.10.10.3.

## Results and discussion

### Pollen production

**Fig 3:**
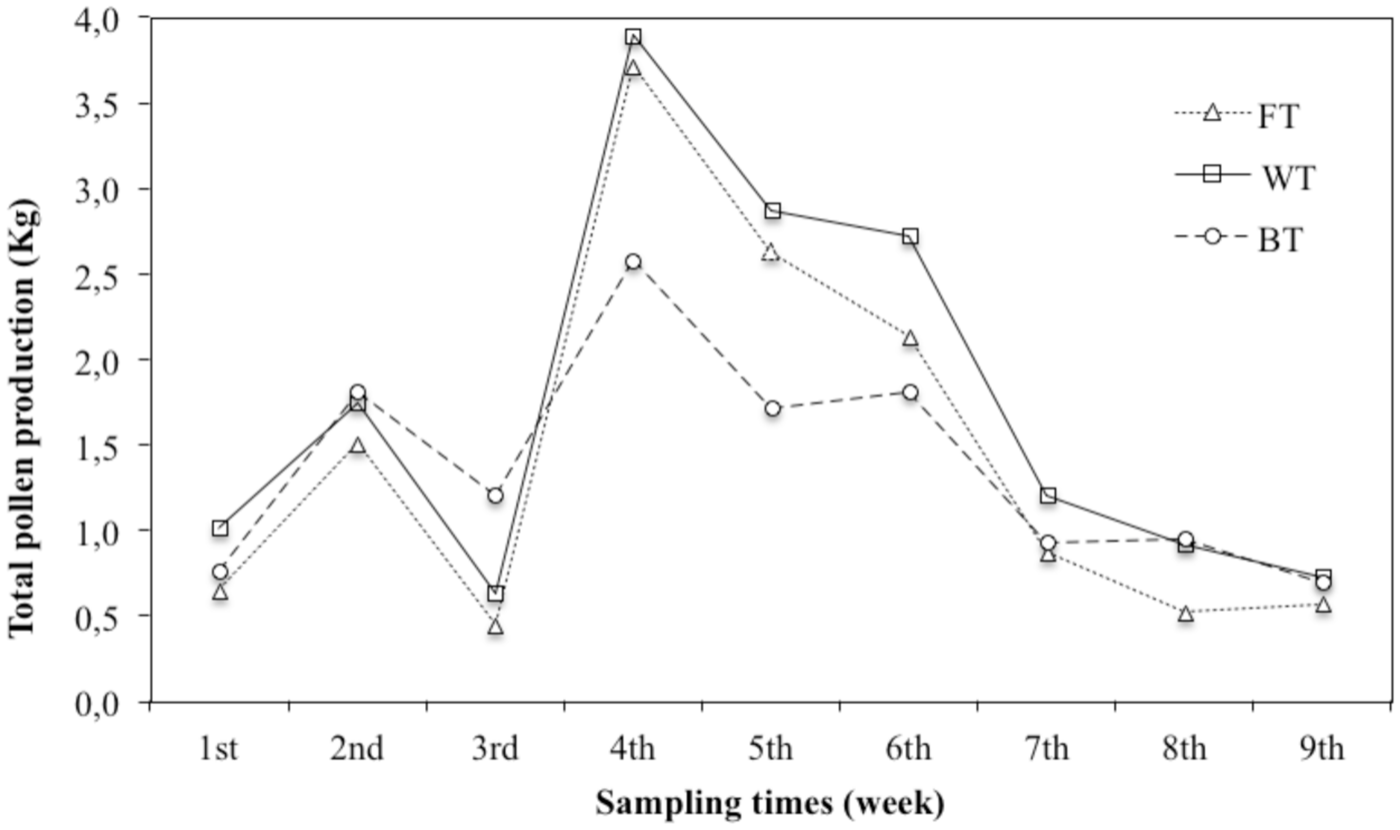
Trends of bee pollen production through 9 weeks for each trap system.

Trend of bee pollen production during the 9 weeks period is reported in Fig 3. The quantity of pollen collected during the whole period was 13.0, 15.8 and 12.5 Kg for FT, WT and BT, respectively. Therefore, FT and BT gave a reduced yield of about 20% with respect to WT. However, the graph shows a similar trend for all traps used, with the maximum yield at 4th sampling point followed by the yields at 5th and 6th sampling point. Noteworthy, about 50% of entire bee pollen production was registered within these 3 weeks. Interestingly, increase of production was registered just after a raining period. It could be related to the low activity of forager bees during raining days and to the consequent their increased activity to recover the pollen supply.

### Results of culture-dependent microbiological analysis on fresh, frozen and dried bee pollen

#### Fresh bee pollen

**Fig 4:**
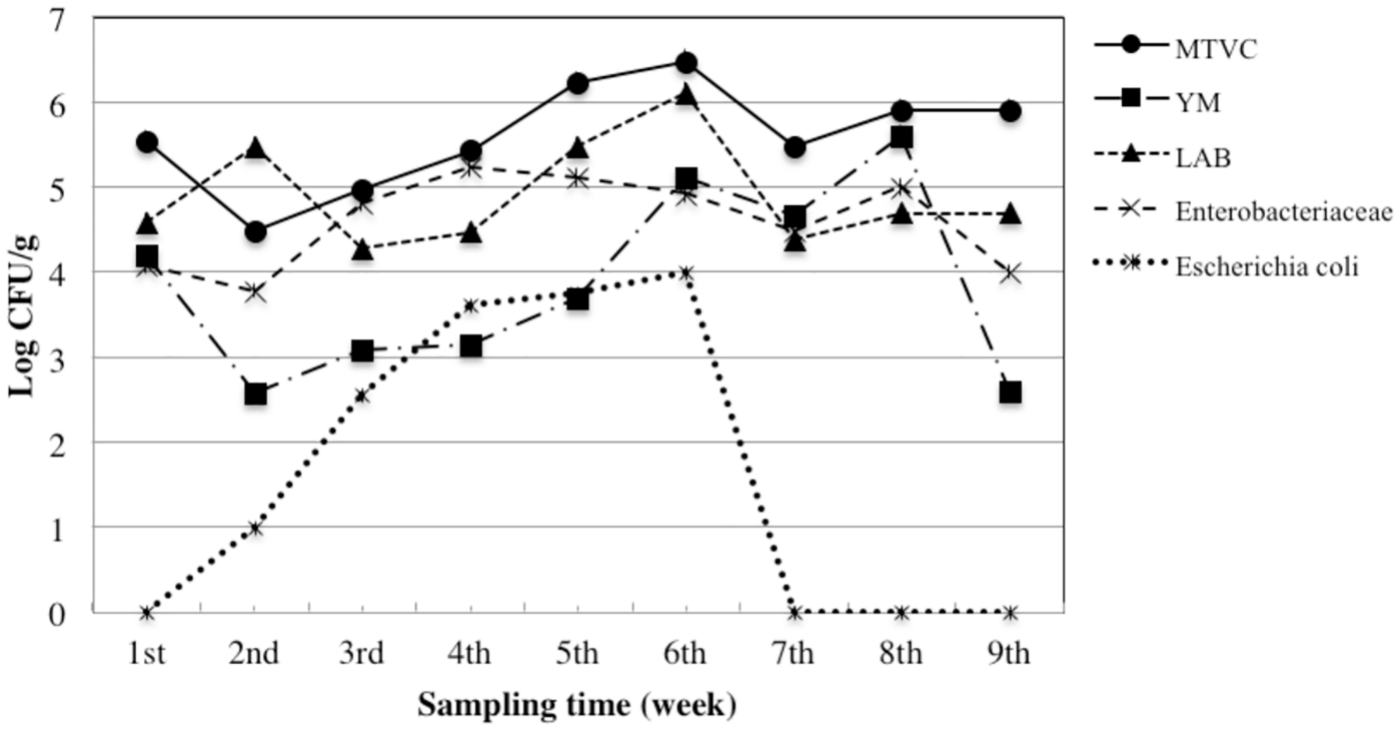
Trends of microbial loads of qualitative parameters in fresh bee pollen collected from hives mounting the front trap. MTVC: mesophilic total viable count; YM: yeast and moulds; LAB: lactic acid bacteria.

**Fig 5:**
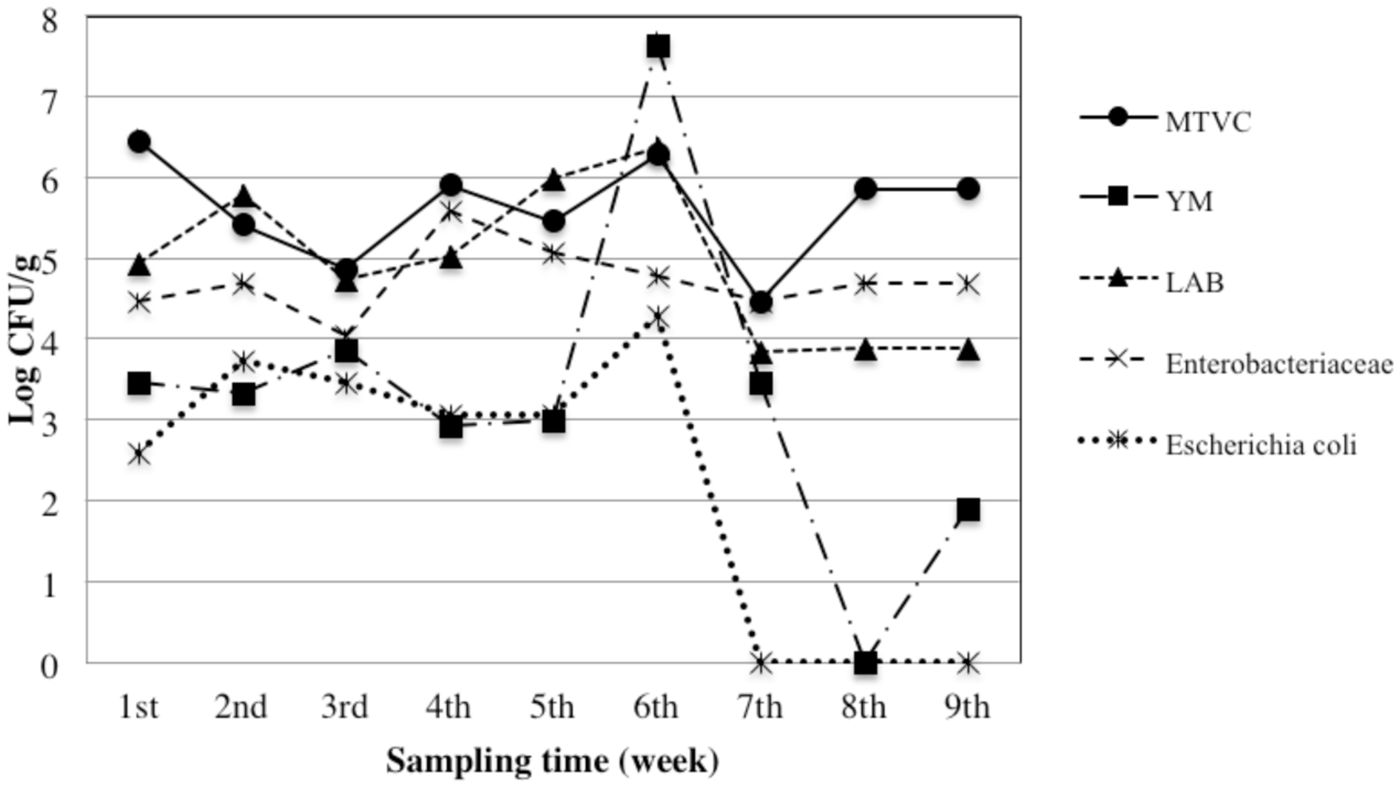
Trends of microbial loads of qualitative parameters in fresh bee pollen collected from hives mounting the wall trap. MTVC: mesophilic total viable count; YM: yeast and moulds; LAB: lactic acid bacteria.

**Fig 6:**
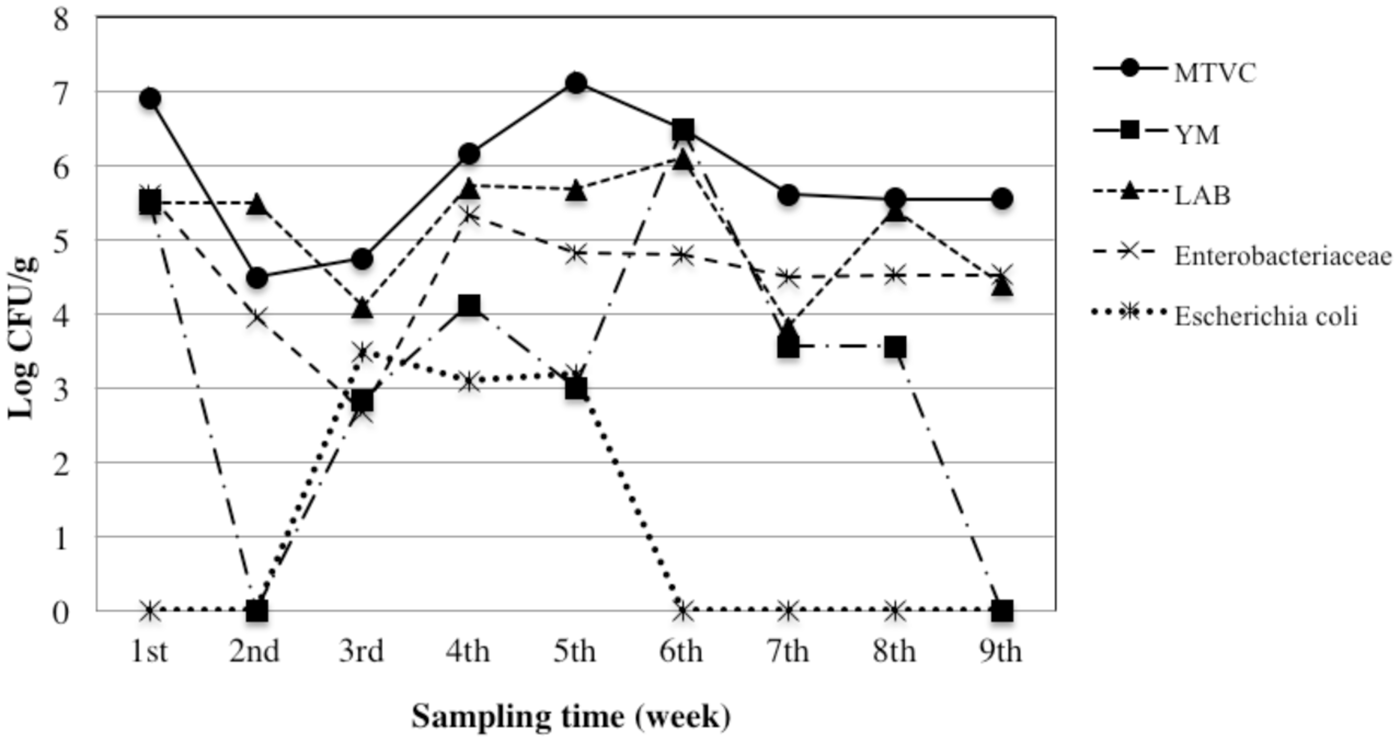
Trends of microbial loads of qualitative parameters in fresh bee pollen collected from hives mounting the bottom trap. MTVC: mesophilic total viable count; YM: yeast and moulds; LAB: lactic acid bacteria.

**Fig 7:**
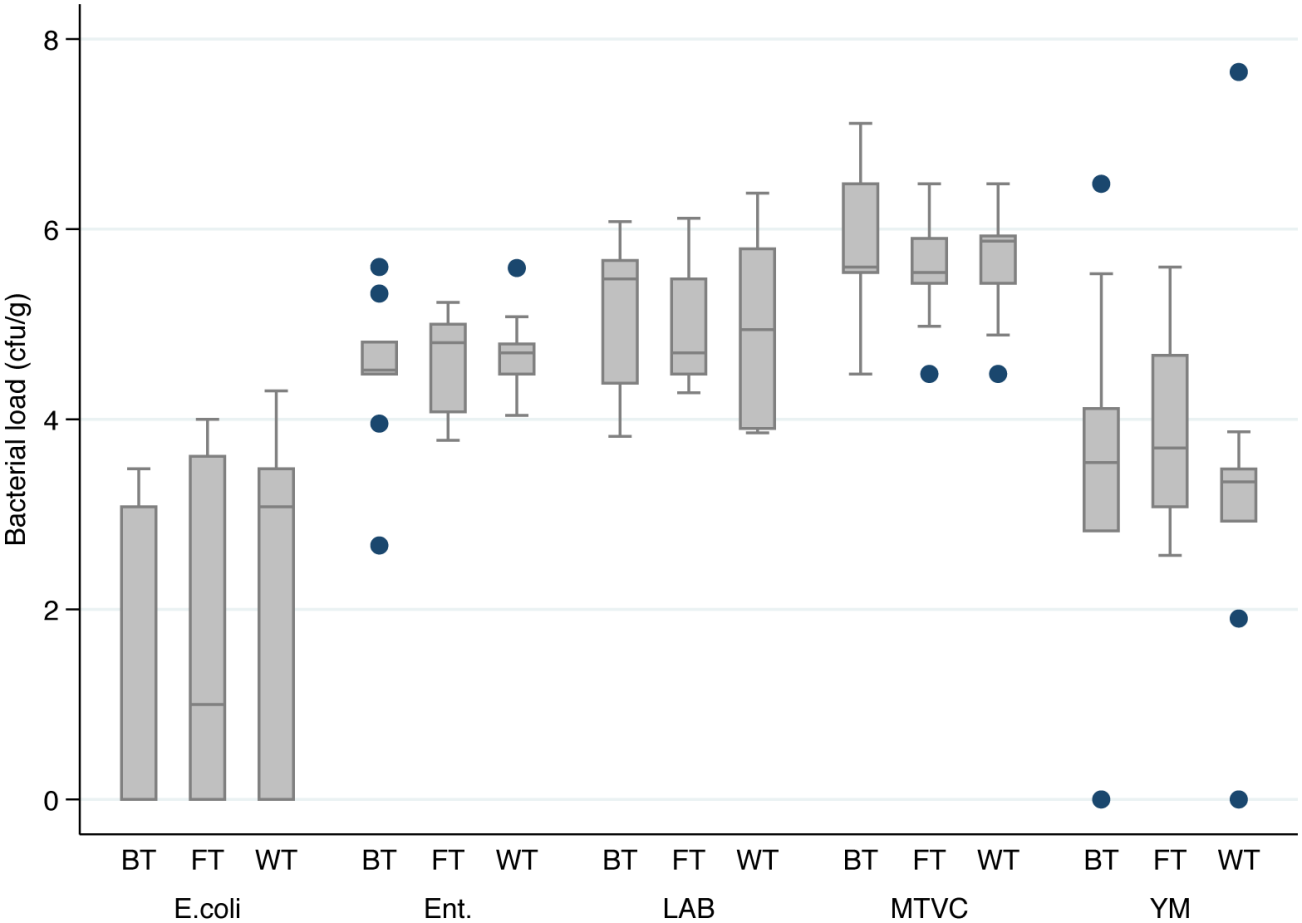
Box plot graph of bacterial load of qualitative microbiological parameters. A one-way Anova analysis showed no significative difference (*p* > 0.05) among traps within the same parameter. BT: bottom trap; FT: front trap; WT: wall trap; E.coli: *Escherichia coli*; Ent.: *Enterobacteriaceae*; LAB: lactic acid bacteria; MTVC: mesophilic total viable count; YM: yeast and moulds.

We took into account both qualitative (MTVC, YM, LAB, *Enterobacteriaceae*, and *E*. *coli*) and safety parameters (*Cl*. *perfringens*, *B*. *cereus*, *S*. *aureus*, *Salmonella* and *L*. *monocytogenes*). Trends of bacterial load for the qualitative parameters are depicted in the Figs 4–6. Moreover, in the Fig 7 is represented a box plot with the total data set registered during the 9 weeks period for each microbial group within the different bee pollen traps. Results for microbiological safety parameters are reported in Table 1.

**Table 1:** Evaluation of microbiological safety parameters (cfu/g for *B*. *cereus* and *S*. *aureus*; presence/absence in 25 g for *Cl*. *perfringens*, *Salmonella* and *Listeria*) of fresh bee pollen samples from hives mounting front trap (FT), wall trap (WT) and bottom trap (BT).

	Parameter	Sampling time								
		1st	2nd	3rd	4th	5th	6th	7th	8th	9th
**FT**	*B*. *cereus*	2.01	4.23	3.98	4.62	u.d.l.^a^	u.d.l.	u.d.l.	u.d.l.	u.d.l.
	*S*. *aureus*	3.00	0.95	2.08	3.95	u.d.l.	3.2	u.d.l.	u.d.l.	u.d.l.
	*Cl*. *perfringens*	-^b^	-	-	+^c^	-	-	-	-	-
	*Salmonella*	+	-	+	-	-	-	-	-	+
	*Listeria*	-	-	-	-	-	-	-	-	-
**WT**	*B*. *cereus*	3.00	4.83	3.04	4.41	u.d.l.	u.d.l.	u.d.l.	u.d.l.	u.d.l.
	*S*. *aureus*	2.02	u.d.l.	2.62	3.57	u.d.l.	u.d.l.	u.d.l.	u.d.l.	u.d.l.
	*Cl*. *perfringens*	-	-	-	-	-	-	-	-	-
	*Salmonella*	-	-	+	-	-	-	-	-	-
	*Listeria*	-	-	-	-	-	-	-	-	-
**BT**	*B*. *cereus*	2.98	3.18	4.30	4.49	2.65	u.d.l.	u.d.l.	u.d.l.	u.d.l.
	*S*. *aureus*	3.78	2.08	2.64	3.08	u.d.l.	3.2	u.d.l.	u.d.l.	u.d.l.
	*Cl*. *perfringens*	-	-	-	-	-	-	-	-	+
	*Salmonella*	-	-	-	-	-	-	-	-	+
	*Listeria*	-	-	-	-	-	-	-	-	-

Values are means of three independent replicates and standard deviation was always less then 1% of mean value.

^a^u.d.l.: under detectable limit of 10 cfu/g.

^b^-: absent in 25 g of pollen.

^c^+: present in 25 g of pollen.

Our results show that the same microbiological qualitative parameter has a similar trend in all types of traps, with slight differences in WT, despite results of one-way Anova analysis, performed considering the single sampling point as a replicated sampling, showed no significant difference (*p*>0.05) in bacterial load for each qualitative parameter among the three different traps (Fig 7).

Generally, the higher contamination level for each bacterial population was found in the samples collected in the middle period (4^th^-6^th^ sampling). As expected, MTVC is the parameter showing higher values in all samples with some exceptions. In fact, higher values than MTVC were registered *i)* for LAB at the end of 2^nd^ week in all traps and at the end of 5^th^ week in WT, *ii)* for YM at the end of 6^th^ week in WT. MTVC ranged between about 4.0 and 6.0 log cycles and trends of all pollen traps showed a peak at 1^st^ and 6^th^ sampling point. On the contrary, lowest bacterial load was registered at 2^nd^ sampling for FT and BT and at 7^th^ sampling for WT. In regard of YM population, only three samples showed absence of colonies, BT at the end of 2^nd^ and 9^th^ week and WT at the end of 8^th^ week, while the maximum value was found at the end of 6^th^ week in WT with 7.6 log cycles. Out of these samples, YM contamination ranged between about 2.5 and 5.5 log cycles with lowest values in the first and last week and higher values at 5^th^ and 6^th^ sampling point. All samples were contaminated by LAB with a bacterial load ranging between about 4.0 and 6.0 log cycles with the maximum level at 6^th^ sampling in all traps. All the samples were contaminated by *Enterobacteriaceae*, whose concentration ranged between 4 and 5 log cycles in most of the samples, with a rather constant trend. Relevant contamination by *E*. *coli* was registered in several samples; in particular it reached about 4 log cycles at 6^th^ sampling for WT. However, in all trap systems *E*. *coli* was under detection limit at 7^th^, 8^th^ and 9^th^ sampling time. Comparing trends of bacterial loads with trends of meteorological parameter it seems that higher level of bacterial loads were especially registered after the raining period. After that, increasing of about 3–4°C in temperature in last period might be related to a general reduction of bacterial load.

Results of safety parameters showed that typical colonies of *B*. *cereus* and *S*. *aureus* were always isolated in the first 4 sampling times (excepted at 2^nd^ sampling time for WT). On the contrary, in the remaining times only few samples showed the presence of these microorganisms. Furthermore, positive result for *Cl*. *perfiringens* was registered only in the samples collected at 4^th^ and 9^th^ week for FT and BT, respectively. Instead, 5 samples, 3 for FT and 1 for BT and WT, showed the presence of typical colonies of *Salmonella*. Finally, typical colonies of *Listeria* were never isolated.

According to our results on viable count of MTVC and LAB, it is clear that most of the bacterial population of bee pollen is constituted by LAB species. Although few works explored the microbial population of bee pollen, our findings are in agreement with results reported by other authors. As a matter of fact, Corby-Harris *et al*. [25] and Anderson *et al*. [26] found, by using a genomic approach, that corbicular bee pollen was mainly contaminated by *Lactobacillus* species and in particular by *Lactobacillus kunkeei*. Unexpectedly, other authors who carried out microbiological analysis of bee pollen were missing to investigate the contamination by LAB. On the other hand, MTVC and YM are the microbiological parameters taken into account for the evaluation of general quality of bee pollen, as reported by Estevinho *et al*. [12], Feás *et al*. [13] and Hani *et al*. [14]. However, only the latter analyzed fresh bee pollen, while early two analyzed bee pollens after a no fully described drying process. Accordingly, our results are in agreement with the findings of Hani *et al*. [14], who reported a contamination level by MTVC and YM of fresh pollen collected in Algeria ranging from 3.0 (collected by hand from date palm) to 6.0 (bee pollen) log cycles. Recently, Nardoni *et al*. [10] investigated the occurrence of potential toxigenic fungi from fresh bee pollen collected in Central Italy. They highlighted the potential risk for human health in bee pollen consumption due to the high contamination level by these moulds. As a matter of fact, Medina *et al*. [27] demonstrated that bee pollen is a substrate that stimulates the production of ochratoxin A by *Aspergillus ochraceus*. A high contamination of *Enterobacteriaceae* in fresh bee pollen was confirmed by the work of Corby-Harris *et al*. [25], who reported high percentage of OTUs belonging to this microbial group. Even the load of coliforms and *S*. *aureus* we found is in agreement with results by Hani *et al*. [14].

#### Frozen bee pollen

Freezing of the samples, collected at the beginning, middle and at the end of collection period, showed to slightly affect microbial composition of pollen for the most of microbiological parameters investigated with no remarkable differences among three trap systems. Results of viable counts of frozen samples from FT, WT and BT during 4 months of storage at -18°C are reported in Tables 2, 3 and 4, respectively. From an overall view, most of the microbiological indicators of pollen quality analyzed were still detected at the end of frozen storage. Samples from the three traps showed after four months of storage an unchanged load for MTVC or never below 4 log CFU/g starting from an average initial load of about 6 log CFU/g. Same trends were found for *Enterobacteriaceae* that recorded an overall reduction of 2 or 3 log cycles from an initial load within 4 and 5.5 log cycles. Also LAB population persisted in samples with a final load between 2.3 and 3.9 log CFU/g after the frozen storage. Interestingly, samples collected at the end of 9^th^ week from all traps showed the most persistent LAB population, which decreased of just 1.5 log cycles with respect to 2.5 log cycles of reduction recorded for all other samples. Based on this result, we hypothesize a different composition of LAB population contaminating bee pollen according to the seasonal period. Noteworthy, the load of yeasts and molds population remained constant or slightly decreased in FT and in BT along the storage while it increased in WT, suggesting, also in this case, a different composition of contaminating population in terms of strains and species, according to the trap system or more probably the position of the hives in the apiary. On the other hand, this condition of storage strongly affected the load of *B*. *cereus*, *S*. *aureu*s and *E*. *coli* population that were not detected at the end of the storage with exception of *B*. *cereus* that was found in the sample collected at the 1^st^ and 4^th^ time of sampling from BT. Furthermore, frozen samples were negative for presence of *Salmonella* spp. even when it was present in the fresh sample indicating that freezing affected *Salmonella* spp. viability. Differently, a typical colony ascribable to *Cl*. *perfringens* was found also after the first month of storage, as confirmed by genotypic analysis, in a sample collected from FT.

**Table 2:** Bacterial load (cfu/g) after 1–4 months of storage at -18°C of samples collected at 1^st^, 4^th^ and 9^th^ time from hives mounting the front trap.

Week of sampling	Microbial parameters	Initial load	Months of storage at -18°C			
			1	2	3	4
1st	MTVC	5.54	4.48	4.48	4.40	4.30
	YM	4.20	3.00	2.93	2.48	2.48
	LAB	4.59	4.30	3.30	3.00	2.30
	*Enterobacteriaceae*	4.08	4.00	3.89	3.00	3.00
	*Bacillus cereus*	2.01	1.00	1.00	u.d.l.	u.d.l.
	*Staphylococcus aureus*	3.00	2.75	2.18	u.d.l.	u.d.l.
	*Escherichia coli*	u.d.l.^a^	u.d.l.	u.d.l.	u.d.l.	u.d.l.
	*Salmonella* spp.	+^b^	-^c^	-	-	-
	*Clostridium perfringens*	-	-	-	-	-
4th	MTVC	5.43	5.24	5.48	5.48	5.2
	YM	3.15	3.12	3.48	3.48	3.00
	LAB	4.48	4.85	4.48	4.48	2.88
	*Enterobacteriaceae*	5.23	5.51	5.60	3.26	2.30
	*Bacillus cereus*	4.62	3.28	3.00	u.d.l.	u.d.l.
	*Staphylococcus aureus*	3.95	2.51	u.d.l.	u.d.l.	u.d.l.
	*Escherichia coli*	3.61	2.95	u.d.l.	u.d.l.	u.d.l.
	*Salmonella* spp.	-	-	-	-	-
	*Clostridium perfringens*	+	+	-	-	-
9th	MTVC	5.90	5.00	5.08	5.48	5.60
	YM	2.60	3.54	3.00	4.88	4.79
	LAB	4.70	4.20	4.30	3.28	3.20
	*Enterobacteriaceae*	5.00	4.70	4.30	4.30	4.30
	*Bacillus cereus*	u.d.l.	u.d.l.	u.d.l.	u.d.l.	u.d.l.
	*Staphylococcus aureus*	u.d.l.	3.00	u.d.l.	u.d.l.	u.d.l.
	*Escherichia coli*	u.d.l	u.d.l.	u.d.l.	u.d.l.	u.d.l.
	*Salmonella* spp.	+	-	-	-	-
	*Clostridium perfringens*	-	-	-	-	-

Values are means of three independent replicates and standard deviation was always less then 1% of mean value.

^a^u.d.l.: under detectable limit of 10 cfu/g.

^b^+: present in 25 g of pollen.

^c^-: absent in 25 g of pollen.

**Table 3:** Bacterial load (cfu/g) after 1–4 months of storage at -18°C of samples collected at 1^st^, 4^th^ and 9^th^ time from hives mounting the wall trap.

Week of sampling	Microbial parameters	Initial load	Months of storage at -18°C			
			1	2	3	4
1st	MTVC	6.48	6.48	6.48	4.51	4.48
	YM	3.40	2.00	3.36	4.48	4.00
	LAB	4.94	u.d.l.^a^	4.36	3.78	3.70
	*Enterobacteriaceae*	4.48	4.00	3.59	3.00	3.00
	*Bacillus cereus*	3.00	2.70	u.d.l.	u.d.l.	u.d.l.
	*Staphylococcus aureus*	2.02	1.54	1.00	3.95	u.d.l.
	*Escherichia coli*	2.61	u.d.l.	u.d.l.	u.d.l.	u.d.l.
	*Salmonella* spp.	-^b^	-	-	-	-
	*Clostridium perfringens*	-	-	-	-	-
4th	MTVC	5.93	5.48	5.48	5.48	5.48
	YM	2.93	3.00	3.30	2.30	3.00
	LAB	5.04	5.40	3.95	3.00	3.11
	*Enterobacteriaceae*	5.59	3.95	3.40	2.30	2.30
	*Bacillus cereus*	4.41	2.83	u.d.l.	u.d.l.	u.d.l.
	*Staphylococcus aureus*	3.57	2.08	u.d.l.	u.d.l.	u.d.l.
	*Escherichia coli*	3.08	2.11	u.d.l.	u.d.l.	u.d.l.
	*Salmonella* spp.	-	-	-	-	-
	*Clostridium perfringens*	-	-	-	-	-
9th	MTVC	5.88	4.90	4.00	4.48	4.60
	YM	1.90	2.54	3.00	4.70	4.70
	LAB	3.90	4.11	4.00	3.28	3.18
	*Enterobacteriaceae*	4.70	4.50	4.30	4.00	3.00
	*Bacillus cereus*	1.00	u.d.l.	u.d.l.	u.d.l.	u.d.l.
	*Staphylococcus aureus*	u.d.l.	u.d.l.	u.d.l.	u.d.l.	u.d.l.
	*Escherichia coli*	u.d.l.	u.d.l.	u.d.l.	u.d.l.	u.d.l.
	*Salmonella* spp.	-	-	-	-	-
	*Clostridium perfringens*	-	-	-	-	-

Values are means of three independent replicates and standard deviation was always less then 1% of mean value.

^a^u.d.l.: under detectable limit of 10 cfu/g.

^b^-: absent in 25 g of pollen.

**Table 4:** Bacterial load (cfu/g) after 1–4 months of storage at -18°C of samples collected at 1^st^, 4^th^ and 9^th^ time from hives mounting the bottom trap.

Week of sampling	Microbial parameters	Initial load	Months of storage at -18°C			
			1	2	3	4
1st	MTVC	6.91	6.48	6.48	5.78	4.48
	YM	5.53	2.00	3.08	2.18	2.30
	LAB	5.48	4.54	4.43	3.28	3.08
	*Enterobacteriaceae*	5.60	5.00	4.88	4.00	4.00
	*Bacillus cereus*	2.98	4.46	3.00	2.78	2.30
	*Staphylococcus aureus*	3.78	3.41	2.92	u.d.l.^a^	u.d.l.
	*Escherichia coli*	u.d.l.	3.54	2.26	u.d.l.	u.d.l.
	*Salmonella* spp.	-^b^	-	-	-	-
	*Clostridium perfringens*	-	-	-	-	-
4th	MTVC	6.15	5.97	5.48	5.48	5.48
	YM	4.11	4.88	4.54	4.30	4.80
	LAB	5.70	4.60	3.60	2.48	2.30
	*Enterobacteriaceae*	5.30	4.25	4.54	3.30	3.30
	*Bacillus cereus*	4.49	3.18	u.d.l.	u.d.l.	4.85
	*Staphylococcus aureus*	3.08	2.04	1.00	u.d.l.	u.d.l.
	*Escherichia coli*	3.08	2.41	u.d.l.	u.d.l.	u.d.l.
	*Salmonella* spp.	-	-	-	-	-
	*Clostridium perfringens*	-	-	-	-	-
9th	MTVC	5.54	5.48	4.90	4.48	4.48
	YM	u.d.l.	3.30	3.00	3.79	3.73
	LAB	4.50	5.08	4.00	4.18	3.95
	*Enterobacteriaceae*	4.52	3.00	3.00	4.48	4.43
	*Bacillus cereus*	3.48	3.00	3.00	u.d.l.	u.d.l.
	*Staphylococcus aureus*	1.00	1.00	u.d.l.	u.d.l.	u.d.l.
	*Escherichia coli*	u.d.l.	1.00	u.d.l.	u.d.l.	u.d.l.
	*Salmonella* spp.	+^c^	-	-	-	-
	*Clostridium perfringens*	+	-	-	-	-

Values are means of three independent replicates and standard deviation was always less then 1% of mean value.

^a^u.d.l.: under detectable limit of 10 cfu/g.

^b^-: absent in 25 g of pollen.

^c^+: present in 25 g of pollen.

To the extent of our knowledge, no previous study aiming the microbiological characterization of frozen bee pollen is available and more in general, no previous work focused on microbial characterization of fresh and frozen Italian bee pollen. Kačániová *et al*. [15] carried out an analysis on Slovakian pollen to see the difference in microscopic fungi and mycotoxin profile in dried, frozen and UV treated pollen concluding that none of these three procedures was efficacious in reducing this kind of contamination in pollen. Further microbiological data on frozen bee pollen were reported in an abstract presented on 2012 to the Annual Meeting of the International Association for Food Protection by Hervatin *et al*. [28]. These authors very concisely highlight the presence of potential pathogens in fresh and frozen Brazilian bee pollen.

#### Dried pollen

Differently from freezing, the dehydration procedure completely affected all microbial communities of bee pollen. Microbiological characterization of dried pollen has been reported in many papers [12, 13] but only in few of them parameters (time and temperature) of dehydrating treatment are reported [15]. Finding from other authors showed that MTVC and YM load of dried pollen collected in different world regions (Portugal, Spain, Brazil, Slovakia) ranged between <10 CFU/g and 10^4^ CFU/g, the level of coliforms was between <1 and 30 UFC/g while *E*. *coli*, *Salmonella*, sulphite-reducing clostridia and *S*. *aureus* were generally absent in all the sample analyzed [12, 13, 29, 30]. The absence of all microorganisms researched in our dried pollen samples could be attributed mainly to the different conditions performed for samples dehydration and also to the diversity in microflora present on pollens of different botanical and geographical origins.

### Molecular identification of pure cultures

Molecular identification based on the amplification of 16S rDNA tract confirmed the presence of some potentially pathogenic microorganisms (Table 5). Only the sequences with a cutoff level of 98% similarity with 16S rDNA sequences available in gene bank have been included in the results. According to the results it was clear that, even though the isolation was carried out on selective media, some bacteria not ascribable to the species investigated in this study have been identified. Several genera of bacteria belonging to *Enterobacteriaceae* family were found and they have been identified as saprophytic species in the environment, reasonably associated to pollen. However, in some cases we cannot univocally attribute the 16S rDNA sequence to a species. Among these some colonies were identified as *Klebsiella oxytoca/Raoultella ornithinolytica/Citrobacter freundii*, all potentially pathogenic microorganisms. For instance, *Klebsiella* and *Citrobacter* have been associated to urinary tract infection [31, 32], while *Roultella ornithinolytica* is associated to aqueous environment, soil, trees and insects and it has been recognized as implicated in cases of human infection [33]. Also the species *Serratia marcescens* was detected in bee pollen samples, and the presence of this opportunistic pathogen has been previously demonstrated by Brindza *et al*. [11] in Slovakian pollen samples.

**Table 5:** Results of phenotypical analysis and molecular identification of typical colonies isolated from bee pollen samples on selective media.

Species attribution (accession number)	Phenotypical characteristics		
	Gram	Catalase	Oxidase
*Bacillus cereus*/*thuringiensis* (MF800964)	+	+	+
*Serratia marcescens/Enterobacter soli/asburiae* (MF801302)	-	+	-
*Enterobacter aerogenes* (MF801304)	-	+	-
*Enterobacter ludwigii/ cancerogenus/ xiangfangensis* (MF860828)	-	+	-
*Escherichia coli/hermanii/Shigella sonnei* (MF801305)	-	+	-
*Klebsiella oxytoca/ Raoultella ornithinolytica/ Citrobacter freundii* (MF801306)	-	-	-
*Cronobacter zurichensis/ Erwinia tasmaniensis* (MF801307)	-	+	-
*Escherichia hermannii/Salmonella enterica* (MF860829)	-	+	-
*Clostridium argentinense* (MF801308)	+	-	-
*Weissella cibaria*/*confusa* (MF801309)	+	-	-
*Staphylococcus gallinarum/succinus* (MF801310)	+	+	-
*Staphylococcus sciuri* (MF801311)	+	+	+
*Staphylococcus saprophyticus/succinus* (MF801312)	+	+	-

Noteworthy, molecular analysis revealed the presence of bacteria identified as *Escherichia*/*Salmonella*. None of the bacteria isolated on BP was identified as *S*. *aureus*, whereas other species of *Staphylococcus* genus were found such as *S*. *saprophyticus*, *S*. *sciuri* and *S*. *gallinarum*, all species classified in the group of coagulase-negative *Staphylococci* and generally considered pathogen of urinary tract. Similar situation occurred for bacteria isolated on SFP since no *Clostridium* identified as *perfringens* was detected. Instead, one isolate was identified in the pathogenic species of *Cl*. *argentinense* and it was found both in fresh and in frozen pollen samples. Molecular analysis also confirmed the presence of *B*. *cereus* species that was present in fresh and frozen pollen.

### High Throughput Sequencing

**Fig 8:**
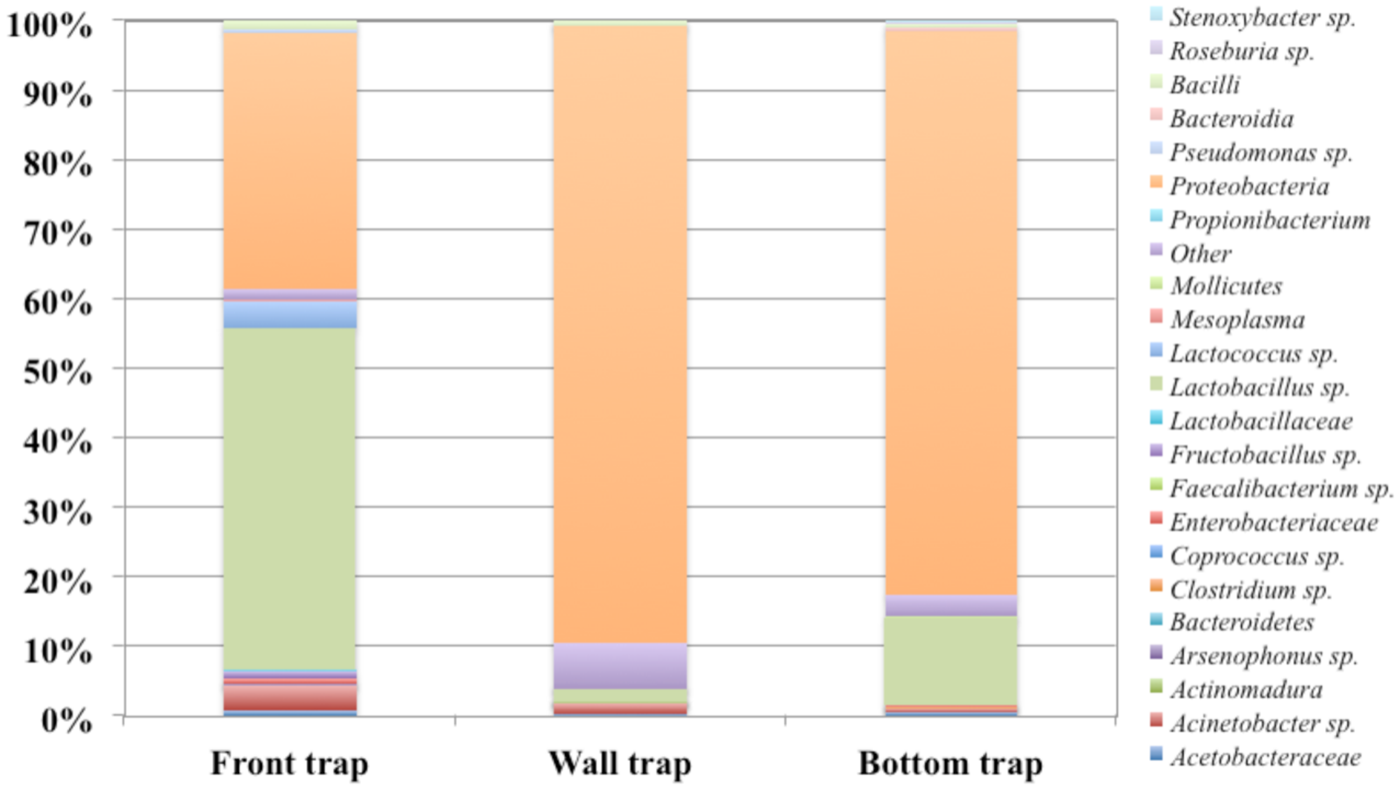
Relative abundance of OUT’s based on 16S rRNA gene by pyrosequencing analysis of DNA from pollen samples.

A total of 4729 raw reads were obtained after 454 processing pipelines. Among which 2749 reads passed the filters applied through QIIME, with an average value of 916 reads/sample. The rarefaction analysis and the estimated sample coverage (Table 6) indicated that there was satisfactory coverage for all samples (ESC above 98% for all the samples). Also, the richness of the samples varied across samples from a minimum of 62 to 95 OUT’s. In Fig 8 all the OTU’s are shown. *Proteobacteria* was the major OTU in BT and WT with 81.3% and 88.6% of relative abundance, respectively. Instead, *Proteobacteria* in FT reaching at 36.7%. *Lactobacillus* sp. occurred only in FT at 49.1%, but at low percentage in BT and WT (12.3% and 1.78% respectively). *Acinetobacter* sp. occurred only in FT and WT at very low percentage (3.7% and 1.5%, respectively), on the other hand *Lactococcus* sp. was found only in FT at 3.9%. The others OUT’s reported in Fig 8 were occurred in all the samples but at low relative abundance < 0.1%. The alpha-diversity index showed a more microbial complexity in FT and WT (Table 6).

**Table 6:** Number of sequences (Reads), observed diversity and estimated sample coverage (ESC) for 16S rRNA amplicons analyzed.

	No. of reads	OTU^a^	Chao1 index	Shannon index	ESC (%)
Front	1132	95	99.75	3.72	98.32
Wall	655	83	83.95	3.71	98.65
Bottom	952	62	71.50	2.31	98.90

Shannon index, Chao1 and Goods coverage were calculated by QIIME at distance level of 3%.

^a^OTU: Operational Taxonomic Units.

## Conclusions

As it can be expected, microbiological analysis of fresh pollen revealed the higher levels of contamination reported until now for all the parameters here investigated in comparison with contamination level of processed pollen. For its nutritional and chemical composition pollen is an ideal matrix for bacterial colonization and growth. Some critical moments in pollen production like the collection and manipulation by beekeepers as well as the storage can favour the spoilage by some microorganisms. Because fresh pollen samples were positive for the presence of some pathogenic species and also levels of MTVC, YM, LAB and *Enterobacteriaceae* were relevant a high health risk could be associated to the consumption of fresh bee pollen. Same assumptions are also referred to consumption of frozen bee pollen while dried pollen remains the safest form of consumption of this high nutritional value product.

Microbiological profile of pollens is a fundamental aspect to guarantee a safe use in human consumption of this product. Many world countries have national standards for pollen, in particular, as reported by Campos *et al*. [1], pollen standards existed at that time in countries such as Brazil, Bulgaria, Poland and Switzerland, while bee pollen has not to be considered like food supplement within EU but like a standard food, hence subjected to standard food legislation. At best of our knowledge this is the first work conducted in Italy on a comprehensive microbiological evaluation of bee pollen, both in its fresh and processed form.

From the results of our analysis it is clear that a more extensive biological hazard analysis should be conducted on bee pollen for human consumption and that biological contamination levels of areas in which hives are positioned should be taken into account from beekeepers.
